# Epstein-Barr virus infection following allogeneic hematopoietic stem cell transplantation in the era of letermovir for cytomegalovirus prophylaxis

**DOI:** 10.1186/s40164-025-00665-0

**Published:** 2025-05-14

**Authors:** Jingtao Huang, Jing Zhou, Shixuan Zhang, Ruoxuan Zhang, Zengkai Pan, Luxiang Wang, Chuanhe Jiang, Jiayu Huang, Zilu Zhang, Yanmin Zhao, Yang Cao, Xiaoxia Hu

**Affiliations:** 1 Shanghai Institute of Hematology, State Key Laboratory of Medical Genomics, National Research Center for Translational Medicine at Shanghai, Ruijin Hospital, Shanghai Jiao Tong University School of Medicine, Shanghai, China; 2 Department of Hematology, Tongji Hospital, Tongji Medical College, Huazhong University of Science and Technology, Wuhan, Hubei China; 3 Bone Marrow Transplantation Center of The First Affiliated Hospital & Liangzhu Laboratory, Zhejiang University School of Medicine, Hangzhou, Zhejiang China

**Keywords:** Allo-HCT, Letermovir, EBV infection, PTLD, Immune reconstitution

## Abstract

Letermovir is an antiviral agent that significantly decreases the frequency of cytomegalovirus (CMV) infections following allogeneic hematopoietic stem cell transplantation (allo-HCT); however, its impact on Epstein-Barr virus (EBV) infection remains unclear. This multicenter, retrospective study involved 565 patients aged ≥ 18 years, who underwent allo-HCT between January 2021 and December 2023, with 284 receiving letermovir prophylaxis (letermovir group) and 281 not (control group). Cumulative incidences of clinically significant CMV infection (cs-CMVi), EBV DNAemia, EBV-disease and post-transplant lymphoproliferative disorder (PTLD) were compared between the groups. The 1-year cumulative incidence of EBV DNAemia did not differ significantly between the letermovir and control groups (58.1% *vs.* 52.7%,* P* = 0.3). However, letermovir prophylaxis was associated with a significantly higher incidence of PTLD within the first year post-HCT (7.39% vs. 1.80%, P = 0.00059). Multivariate analysis identified letermovir prophylaxis as an independent risk factor for PTLD (HR [95% CI]: 4.619 [1.458–10.278], P = 0.007). Letermovir altered the early reconstitution trajectory after allo-HCT, particularly in CD8^+^ T cells. Our findings emphasized that although letermovir prophylaxis did not increase the risk of EBV DNAemia in allo-HCT recipients, it was associated with a higher incidence of PTLD. Further studies focusing on immune reconstitutiom dynamics are warranted to elucidate the underlying pathophysiology of EBV-PTLD under letermovir pressure.

**Supplementary Information:**

The online version contains supplementary material available at 10.1186/s40164-025-00665-0.

To the Editor,

Epstein-Barr virus (EBV) reactivation affects 19.6–65.0% of individuals following allogeneic hematopoietic stem cell transplantation (allo-HCT) [1–3], and is closely associated with post-transplant lymphoproliferative disorder (PTLD) [4, 5]. Letermovir is an antiviral agent that significantly decreases cytomegalovirus (CMV) reactivation following allo-HCT [6], but its impact on EBV infections remains unclear.

To address this issue, we performed a retrospective multicenter study to explore the association of letermovir prophylaxis with EBV reactivations in allo-HSCT recipients. 706 allo-HCT recipients were screened, with 565 patients eligible for further analysis (Fig. 1A and Supplementary Methods). Stratified by letermovir use, patients were grouped into the letermovir group (n = 284) and the control group (n = 281) (Supplementary Table 1). The median letermovir exposure was 100 (range: 54–327) days (Supplementary Fig. 1 and Table 1).

**Fig. 1:**
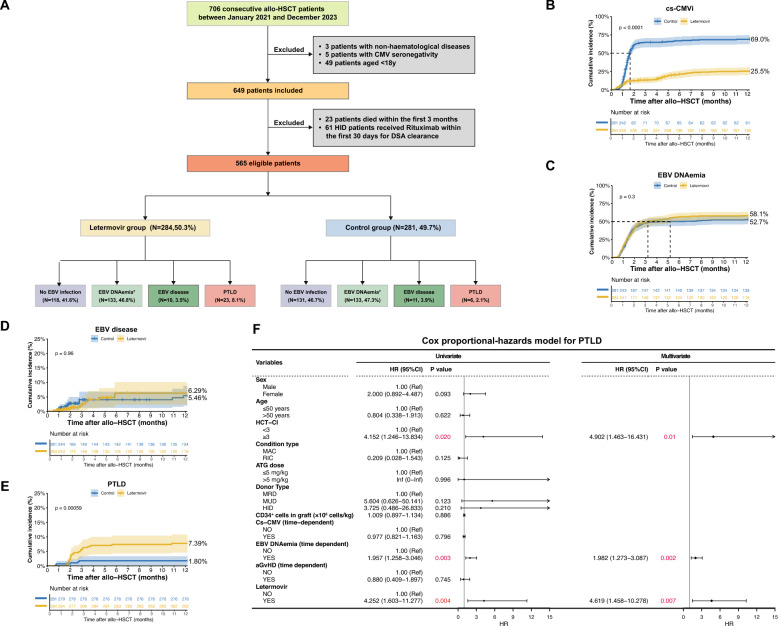
The impact of letermovir prophylaxis on EBV infections after allo-HCT.** A** Study flowchart. The cumulative incidences of cs-CMVi (**B**), EBV DNAemia (**C**), EBV-disease (**D**) and PTLD (**E**) in the letermovir and control groups, respectively. **F** Cox proportional-hazards analysis for EBV reactivation. DSA: donor-specific antibodies; HID: haploidentical donor; MRD: HLA-matched related donor; MUD: HLA-matched unrelated donor. *EBV DNAemia refers to those patients without EBV-disease/PTLD development

Letermovir reduced the 1-year cumulative incidence of clinically significant CMV infection (cs-CMVi) (25.5% *vs.* 69.0%, *P* < 0.0001; Fig. 1B) and delayed the occurrence of cs-CMVi (median [range]: 104 [14–336] *vs.* 39 [13–330] days, *P* < 0.0001; Supplementary Table 1) compared to the control group. There was no significant difference in the cumulative incidences of EBV DNAemia (58.1% *vs.* 52.7%,* P* = 0.3; Fig. 1C) and EBV-disease (6.29% *vs.* 5.46%, *P* = 0.96; Fig. 1D) between the letermovir and control groups during the first year. Antecedent EBV DNAemia was documented in all the patients with EBV-disease (n = 21) or PTLD (14 patients with proven PTLD and 15 with probable PTLD). The 1-year cumulative incidence of PTLD was significantly higher in the letermovir group than the control group (7.39% *vs.* 1.80%, *P* = 0.00059; Fig. 1E). Detailed information about the clinical characteristics of patients with EBV-disease/PTLD is shown in Supplementary Table 2 and 3. Multivariate Cox analysis identified high hematopoietic-cell-transplantation-specific comorbidity index (HCT-CI) score (≥ 3), precedent EBV DNAemia, and letermovir are risk factors for PTLD (Fig. 1F).

Considering that letermovir may alter the dynamics of Immune reconstitution (IR) post-transplant [7, 8], we next quantified IFN-γ release in peripheral blood mononuclear cells (PBMCs) from 83 letermovir-treated patients to assess antiviral capacity. We found that IFN-γ release peaked earlier in patients with EBV DNAemia or EBV-disease than in patients without EBV infections, but remained impaired in PTLD cases (Fig. 2A). The partial least squares discriminant analysis (PLS-DA) revealed that PTLD patients exhibited a distinct lymphocyte reconstitution pattern from other groups (Fig. 2B), especially delaying CD8^+^ T-cell recovery (Fig. 2C and Supplementary Fig. 2A). These findings suggested similar IR kinetics between EBV-disease and EBV DNAemia. Due to a small number of evaluable EBV-disease cases (n = 4), the two groups were pooled for subsequent analyses.

**Fig. 2:**
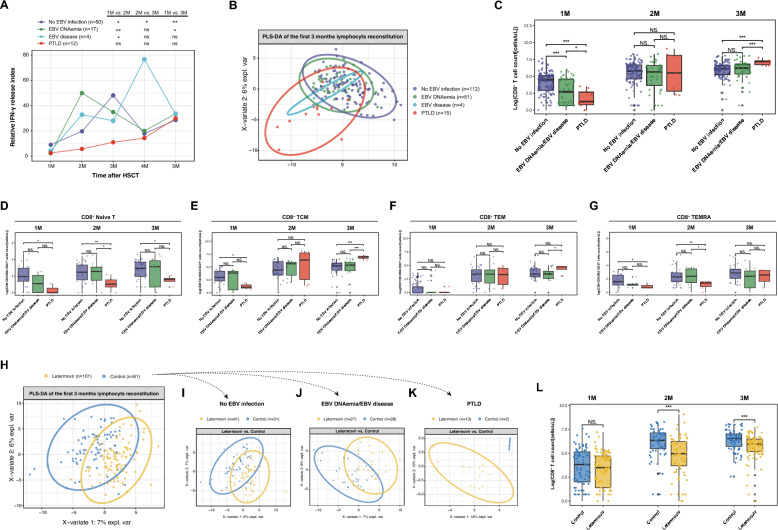
Immune reconstitution related EBV infections and letermovir prophylaxis following allo-HCT. Analysis of the relative IFN-γ release ability by PBMCs in different groups at different timepoints following allo-HCT (**A**); Results of the PLS-DA, showing lymphocyte recovery in the first 3 months after allo-HCT in patients with no EBV infection, EBV DNAemia (without subsequent PTLD, thereafter)/EBV-disease and PTLD, respectively (**B**); In comparison to patients without EBV infection, CD8^+^ T cell recovery was progressively impaired in patients with DNAemia/EBV-disease and PTLD (no EBV infection > DNAemia/EBV-disease > PTLD) in the first month after allo-HCT (**C**); Comparison of naïve CD8^+^ T (T_N_, CD8^+^CD45RA^+^CD27^+^) (**D**), central memory CD8^+^ T (T_CM_, CD8^+^CD45RA^−^CD27^+^) ** (E)**, effector memory CD8^+^ T (T_EM_, CD8^+^CD45RA^−^CD27^−^) (**F**), and terminally differentiated T_EM_ (T_EMRA_, CD8^+^CD45RA^+^CD27^−^) CD8^+^ T cells (**G**) among patients with no EBV infection (n = 33), EBV DNAemia/EBV-disease (n = 25), or EBV-disease/PTLD (n = 6); Results of the PLS-DA, showing lymphocyte recovery in the first 3 months following allo-HCT in the letermovir and control groups, respectively (**H**); Results of the PLS-DA, showing lymphocyte recovery in patients without EBV infection (**I**), patients with EBV DNAemia/EBV-disease (**J**), or PTLD patients (**K**) in the first 3 months following allo-HCT in the letermovir and control groups, respectively; The effect of letermovir prophylaxis on the CD8^+^ T cell recovery trajectory (**L**). *P < 0.05, **P < 0.01, ***P < 0.001. Data about lymphocyte count are presented after logarithmic conversion. ‘Patients with EBV DNAemia’ refers to those patients without subsequent EBV-disease/PTLD occurrences

Naïve CD8^+^ T (T_N_) cells were deficient in PTLD patients during the first 3 months after allo-HCT (Fig. 2D). The frequencies of central memory CD8^+^ T (T_CM_) and effector memory CD8^+^ T (T_EM_) cells were comparable in all patients at the 2nd month (Fig. 2E, F), while the terminally differentiated T_EM_ (T_EMRA_) cells were decreased in PTLD patients during the same period (Fig. 2G). We hypothesized that letermovir may impede the immune recovery in certain patients who exhibit poor IR post-transplant, potentially leading to severe EBV infection (i.e., EBV-PTLD).

As expected, letermovir prophylaxis altered the reconstituted immune repertoire relative to that of the control group (Fig. 2H) and the impact on IR was progressively amplified across patients without EBV infection, patients with EBV DNAemia/disease, and PTLD patients (Fig. 2I–K). The delayed recovery pattern also extended to multiple CD8^+^ T-cell subsets (Supplementary Fig. 2B). Although the CD8^+^ T-cells frequencies showed no significant differences during the 1st month after allo-HCT, letermovir recipients exhibited impaired reconstitution of these cells in the 2nd and 3rd months compared to controls (Fig. 2L).

In this study, we identified letermovir as an independent risk factor for PTLD, significantly influencing the early IR pattern after allo-HCT. This effect was mediated primarily by impaired CD8^+^ T-cell recovery. PTLD patients had markedly few CD8^+^ T_EMRA_ cells, characterized by reduced proliferative potential but strong cytotoxic and proinflammatory activities [9]. CD27^+^EBV-specific cytotoxic CD8^+^ T cells are pivotal in controlling EBV [10]. We also identified two CD27-expressing T subsets (CD8^+^ T_N_ and T_CM_ cells) in PTLD patients with letermovir prophylaxis that showed impaired recovery before PTLD onset. Typically, viral antigen exposure activates CD8^+^ T_N_ cells, transforming them into cytotoxic T lymphocytes, through T cell receptor-dependent and -independent mechanisms [11, 12]. Thus, letermovir prophylaxis may impair early post-transplant CD8^+^ T_N_ cell reconstitution and further differentiation, compromising EBV DNAemia control and increasing PTLD risk.

In conclusion, letermovir prophylaxis was associated with a higher incidence of PTLD after allo-HCT. Our findings emphasized the significance of early CD8^+^ T-cell recovery, which may help to predict the outcomes of EBV insfections in the letermovir era. Although the retrospective nature of this study prevented us from delineating the underlying mechanisms, we hope the relationship between letermovir and EBV infections will be further explored in prospective cohort and translational studies.

## Supplementary Information

Supplementary material 1.

## Data Availability

The datasets generated and analysed in the present study are available from the corresponding author (hu_xiaoxia@126.com) upon reasonable request.

## Abbreviations

Allo-HCT: Allogeneic hematopoietic stem cell transplantation
CMV: Cytomegalovirus
Cs-CMVi: Clinically significant CMV infection
EBV: Epstein-Barr virus
IR: Immune reconstitution
PLS-DA: Partial least squares discriminant analysis
PTLD: Post-transplant lymphoproliferative disorder
