# Supplementary material for: Epstein-Barr virus infection following allogeneic hematopoietic stem cell transplantation in the era of letermovir for cytomegalovirus prophylaxis
Source: Exp Hematol Oncol. 2025 May 14;14:72. doi: 10.1186/s40164-025-00665-0 (PMC12079889; doi:10.1186/s40164-025-00665-0)
Supplement: Supplementary file 1 — Supplementary material 1. [file 40164_2025_665_MOESM1_ESM.docx]

**Supplementary materials**

**Epstein-Barr virus infection following allogeneic hematopoietic stem cell transplantation in the era of letermovir for cytomegalovirus prophylaxis**

**Authors：**Jingtao Huang^1,4^, Jing Zhou^2,4^, Shixuan Zhang^3,4^, Ruoxuan Zhang^2,4^, Zengkai Pan^1^, Luxiang Wang^1^, Chuanhe Jiang^1^, Jiayu Huang^1^, Zilu Zhang^1^, Yanmin Zhao^#,3^, Yang Cao^#,2^, Xiaoxia Hu^#,1^

**Affiliations:**

^1^ Shanghai Institute of Hematology, State Key Laboratory of Medical Genomics, National Research Center for Translational Medicine at Shanghai, Ruijin Hospital, Shanghai Jiao Tong University School of Medicine, Shanghai, China

^2^ Department of Hematology, Tongji Hospital, Tongji Medical College, Huazhong University of Science and Technology, Wuhan, Hubei, China

^3^ Bone Marrow Transplantation Center of The First Affiliated Hospital & Liangzhu Laboratory, Zhejiang University School of Medicine, Hangzhou, Zhejiang, China

^4^ The authors contribute equally.

**^#^Correspondence:** Xiaoxia Hu: hu_xiaoxia@126.com; Yang Cao: caoyangemma@163.com; Yanmin Zhao: yanminzhao@zju.edu.cn

Contents

[Supplementary Methods - 3 -](#_Toc191072040)

[**Study design and patients** - 3 -](#_Toc191072041)

[**Data collection** - 3 -](#_Toc191072042)

[**Transplantation procedures** - 4 -](#_Toc191072043)

[**Definitions** - 4 -](#_Toc191072044)

[**Prophylaxis and preemptive therapy for virus reactivation** - 5 -](#_Toc191072045)

[**Preemptive therapy for EBV infections** - 6 -](#_Toc191072046)

[**Interferon-γ (IFN-γ) release assay** - 6 -](#_Toc191072047)

[**Immune reconstitution monitoring** - 7 -](#_Toc191072048)

[**Statistical analysis** - 7 -](#_Toc191072049)

[Supplementary Figures - 9 -](#_Toc191072050)

[**Supplementary Figure 1. The administration of letermovir prophylaxis to allo-HCT recipients treated at each centre.**. - 9 -](#_Toc191072051)

[**Supplementary Figure 2. The weights of lymphocyte subsets reconstituted within the first 3 months after allo-HCT contribute to the different IR patterns.**. - 10 -](#_Toc191072052)

[Supplementary Tables - 11 -](#_Toc191072053)

[**Supplementary Table 1. Patient characteristics** - 11 -](#_Toc191072054)

[**Supplementary Table 2. Clinical characteristics of patients with EBV-disease** 14](#_Toc191072055)

[**Supplementary Table 3. Clinical characteristics of patients with PTLD** 15](#_Toc191072056)

[References 17](#_Toc191072057)

**Supplementary Methods**

**Study design and patients**

This multicenter, retrospective study was designed by the First Affiliated Hospital (Zhejiang University School of Medicine), the Wuhan Tongji Hospital, and the Shanghai Ruijin Hospital. The study enrolled CMV-seropositive patients who received the first allo-HCT at the three transplant centers prior to (January 2021 to July 2022) and after (July 2022 to December 2023) the routine use of letermovir for CMV prophylaxis. The eligibility criteria were as follows: (1) aged ≥ 18 years and with hematological diseases; (2) undergoing the first allo-HCT and have a life expectancy ≥ 3 months; (3) allo-HCT recipients with CMV seropositivity; (4) complete follow-up information. Considering that anti-CD20 antibodies may diminish the probability of EBV infections (1, 2), patients who received anti-CD20 antibodies for the desensitization of donor-specific anti-HLA antibodies (DSA) within the first 30 days after allo-HCT were excluded(3, 4). The last follow-up visit took place on March 31, 2024. The study was approved by the Institutional Review Board of each participating hospital and was conducted in accordance with the *Declaration of Helsinki* and its amendments.

**Data collection**

Investigators at each hospital used a chart review, institutional electronic medical records, and clinical databases to obtain the required data. The collected data included information on patient demographics (e.g., recipient and donor CMV and EBV serostatus), underlying diseases, transplant procedures (e.g., conditioning regimen and GvHD prophylaxis modalities), CMV infection (e.g., cs-CMVi), EBV infections (e.g., involved organs), PTLD, aGvHD severity, letermovir treatment (e.g., the administration method, dosage, and duration of the medication), and long-term clinical outcomes (e.g., mortality and survival). The data were independently reviewed by two physicians with rich expertise in allo-HCT.

**Transplantation procedures**

The stem cell sources included granulocyte-stimulating-factor-mobilized peripheral blood stem cells from HLA-matched related donors (MRDs, n=74), HLA-matched unrelated donors (MUDs, n=54) and haploidentical donors (HIDs, n=437). The major myeloablative condition (MAC) regimens included fludarabine, busulfan, and melphalan (FBM) and busulfan plus cyclophosphamide (BuCy), which have been previously described(5-7). Reduced-intensity conditioning (RIC) consisted of fludarabine and busulfan (6.4 mg/kg total dose, administered intravenously). 476 (84.2%) and 89 (15.8%) patients underwent MAC and RIC, respectively.

Acute graft versus host disease (aGvHD) prophylaxis mainly consisted of anti-thymocyte globulins (ATG) and post-transplant cyclophosphamide (PTCy), as previously reported(6, 8). A total of 184 adult allo-HCT recipients with HID grafts, who were enrolled in a prospective clinical trial for aGvHD prophylaxis (NCT 03608059)(9), were included in the present study. The ATG + PTCy regimen consisted of 2.5 mg/kg/day ATG administered on days −2 to −1, and 50 mg/kg cyclophosphamide administered on day +3. Patients who received grafts from MRDs received cyclosporine A, short-term methotrexate, and mycophenolate mofetil following stem cell infusion. The trough concentrations of calcineurin inhibitors were maintained according to standard clinical practices (cyclosporine: 200-300 ng/mL; tacrolimus: 6-10 ng/mL). If a MUD was deemed acceptable for allo-HCT, ATG was incorporated into the aGvHD prophylaxis regimen. The infection prophylaxis protocols have been previously published(10).

**Definitions**

CMV and EBV loads were measured at the department of laboratory medicine of each participating centre using ﻿real-time quantitative polymerase chain reaction (RT-qPCR). The PCR primer sequences used for the amplification and detection of CMV and EBV genes have been previously reported(11-13). Weekly CMV and EBV monitoring was performed in the first 3 months after allo-HCT; bi-weekly monitoring until 6 months after allo-HCT was then performed at outpatient visits. Clinically significant CMV infection (cs-CMVi) included cs-CMV DNAemia necessitating preemptive therapy and/or CMV disease.

EBV DNAemia was diagnosed when the plasma EBV DNA loads ≥ 5 × 10^2^ IU/mL at any time. EBV-disease/PTLD was diagnosed according to the World Health Organization (WHO) classification of lymphoid neoplasms (2023 revision)(14) and the Sixth European Conference on Infections in Leukemia (ECIL-6) guidelines(15, 16). The diagnosis of EBV-disease was based on symptoms and/or signs involving only the extranodal sites, along with the biopsy. PTLD ﻿was categorized as "proven PTLD" and "probable PTLD". Proven PTLD was characterized according to the clinical manifestations (fever, lymphadenopathy, hepatosplenomegaly, or other extranodal involvement) and published pathological diagnostic criteria(17). For patients who could not undergo a biopsy but had the corresponding clinical manifestations, probable PTLD was considered if clinical manifestations with significant EBV DNAemia were present and other causes were ruled out(15). According to the definitions, 29 patients with PTLD were categorized as EBV-PTLD (14 with proven and 15 probable). In situ hybridization confirmed that all fourteen patients with proven PTLD were positive for EBV-encoded small RNA (EBER).

**Prophylaxis** **and preemptive therapy for virus reactivation**

Allo-HCT recipients who did not receive letermovir prophylaxis (n=281) were given ganciclovir at a dose of 5 mg/kg twice daily for 7-10 days before graft infusion. After the application of letermovir in July 2022, allo-HCT recipients (n=284) received a dose of 480 mg/day (or 240 mg/day if receiving concomitant cyclosporine) of letermovir before neutrophil engraftment until day 100. The duration of letermovir prophylaxis is beyond day 100 for patients with acute/chronic GvHD necessitating immunosuppressive therapy according to clinical practice. For patients with gastrointestinal intolerance, letermovir was given intravenously instead of orally.

Preemptive therapy (with ganciclovir, valganciclovir, and/or foscarnet) was initiated in cases of cs-CMVi at the discretion of the treating physician. Because of inaccessibility to cidofovir across the three transplant centres, cidofovir was not included in the preemptive therapy or primary prophylaxis regimens for CMV management. Once the cs-CMVi was confirmed, preemptive therapy, comprising ganciclovir, valganciclovir, and foscarnet, was initiated.

All patients received general infection prophylaxis for herpes simplex virus (HSV) and varicella-zoster virus (VZV) reactivation following allo-HCT(18-20). Acyclovir (400 mg, twice a day) was offered to all HSV-seropositive allo-HCT recipients to prevent HSV and VZV reactivation at the start of conditioning therapy. Recipients of grafts from MRDs were given acyclovir for at least 6 months after allo-HCT. Recipients of grafts from MUDs or HIDs were given acyclovir for ≥1 year or until their absolute CD4^+^ T cell counts in the peripheral blood reached ≥ 200/μL during immunosuppressive therapy tapering.

**Preemptive therapy for EBV infections**

Patients with plasma EBV DNA loads ≥ 2 × 10^3^ IU/mL at any time, or two consecutive plasma EBV DNA loads ≥ 1 × 10^3^ IU/mL within 1 week, or with symptoms and/or signs attributable to EBV DNAemia and EBV-disease/PTLD (adjudicated by the treating physician), were initiated on preemptive therapy(16). The primary preemptive therapy mode was rituximab administration (dose 375 mg/m^2^, once weekly until EBV DNAemia negativity was achieved), as well as a reduction in the degree of immunosuppression.

**Interferon-γ (IFN-γ) release assay**

The antiviral immune response of the allo-HCT recipients was evaluated using the FlowSpot assay as previously described(21). Briefly, PBMCs were stimulated with 3 μg/mL of the mitogen phytohemagglutinin (positive control), while the unstimulated cells were used to measure background cytokine production (negative control). Subsequently, 1 × 10^5^ freshly isolated PBMCs were co-cultured with IFN-γ-specific capture beads (BD technologies, USA) at 37 ℃ and 5% CO_2_ for 16 hours in triplicate (approximately 7000 beads per replicate). After incubation, the capture beads were sorted and then incubated with a phycoerythrin-conjugated secondary antibody for 2 hours at room temperature. The capture beads were then analysed using a BD FACSCanto II flow cytometer after two rounds of washing with 150 μL wash buffer. The relative IFN-γ release index was calculated as the ratio of the positive to the negative signal.

**Immune reconstitution monitoring**

Flow cytometry was used to analyse lymphocyte reconstitution at months 1, 2, and 3 after allo-HCT. A total of 182 patients from the three centres were included in this part of the analysis. The reporting of these data was approved by the Institutional Review Board (IRB) of each participating hospital (IRB number: 2023-349).

After neutrophil engraftment (≥ 500 cells/μL), various lymphocyte subsets were characterised by flow cytometry at months 1, 2, and 3 after allo-HCT. Cell acquisition was performed on a FACSCanto II flow cytometer (BD Biosciences). All antibodies were optimized for performance, and appropriate single-color compensation and Institutional Review Board (IRB) controls were used. A time gate was initially established to ensure consistent sample collection. Lymphocytes were identified by gating on “live” (Brilliant Violet 510^−^), CD14^−^ (to exclude monocytes) cells with appropriate size (FSC) and granularity (SSC) characteristics. Data were analysed with FlowJo software, version 10.8.1.

**Statistical analysis**

Chi-squared or Fisher’s exact tests were used to compare different categorical variables between the two groups. Continuous variables were compared using the unpaired two-tailed Student’s t-test or the Welch's t-test (for two-group comparisons) or a one-way ANOVA (for multiple-group comparisons). Kaplan–Meier method and log‐rank test were used to determine the cumulative incidence of cs-CMVi, EBV DNAemia, and EBV-disease/PTLD.

A Cox regression model with time-varying covariates was used to determine risk factors for PTLD. Multivariate Cox regression analysis was performed on variables with a P-value < 0.05 identified in the univariate analysis.

To characterize the IR trajectory of each patient in the first 3 months after allo-HCT, partial least squares discriminant analysis (PLS-DA) was performed using the “mixOmics” R package. Correlation analysis was performed using the R package “corrplot”.

A *P-*value < 0.05 was considered as a measure of statistical significance. All analyses were performed using R software version 4.3.1.

**Supplementary Figures**

**
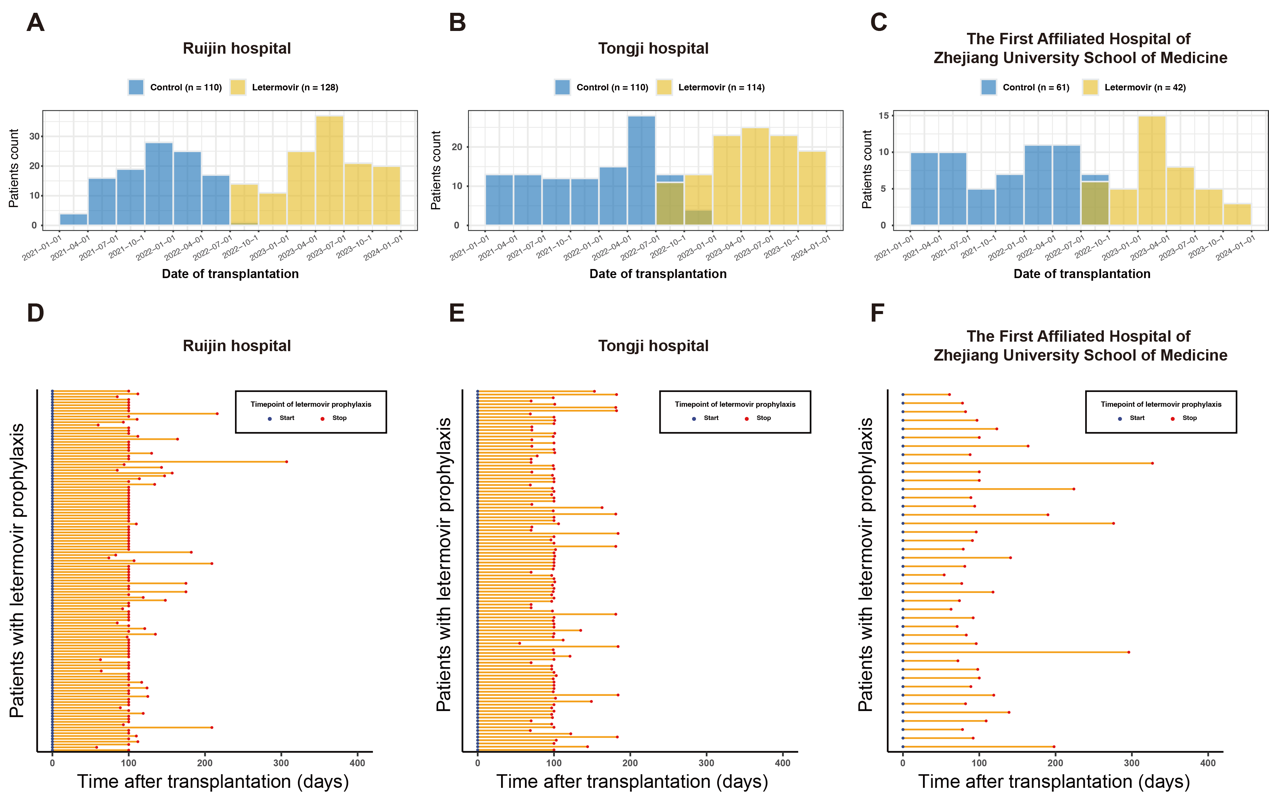
**

**Supplementary Figure 1. The administration of letermovir prophylaxis to allo-HCT recipients treated at each center.** The time distribution of allo-HCT date for the letermovir and control groups at the Ruijin Hospital **(**n = 238, **A)**, the Tongji Hospital **(**n = 224, **B)**, or The First Affiliated Hospital, Zhejiang University School of Medicine **(**n = 103, **C)**. The letermovir maintenance time of patients treated at the Ruijin Hospital **(**n = 128, **D)**, the Tongji Hospital **(**n = 114, **E)**, or The First Affiliated Hospital, Zhejiang University School of Medicine **(**n = 42, **F)**.

**
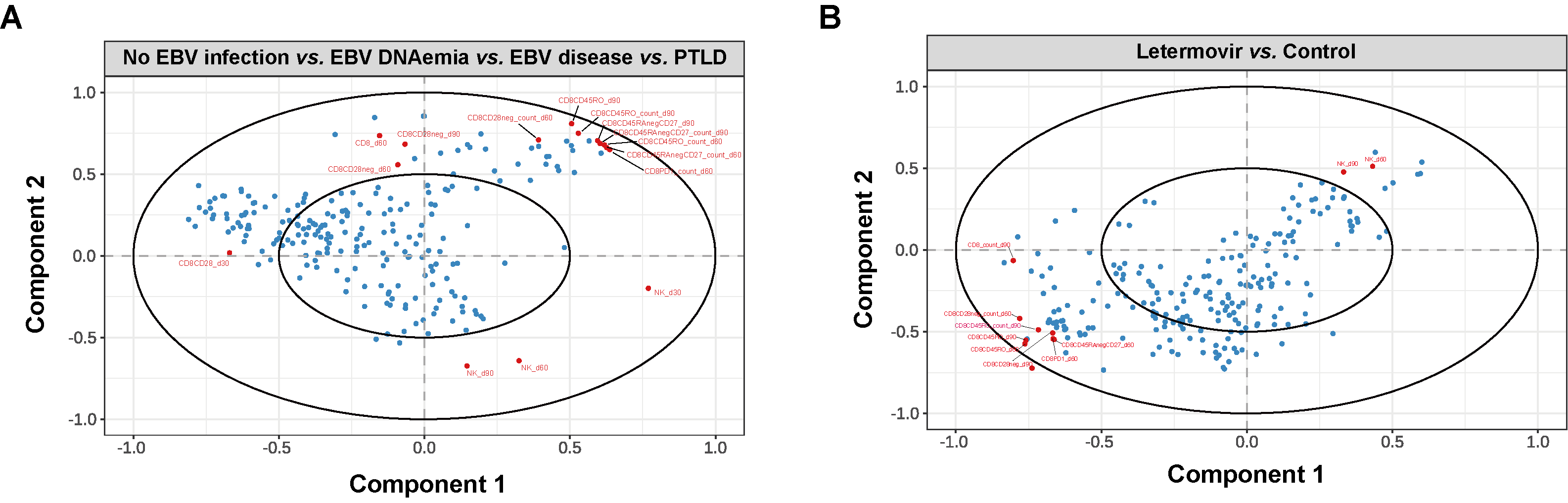
**

**Supplementary Figure 2. The weights of lymphocyte subsets reconstituted within the first 3 months after allo-HCT contribute to the different IR patterns. (A)** Comparison of IR patterns among patients with no EBV infection, EBV DNAemia, ENV-disease, or PTLD. **(B)** Comparison of IR patterns between patients in the letermovir group (n = 101) and the control group (n = 81). ‘Patients with EBV DNAemia’ refers to patients without subsequent EBV-disease/PTLD.

**Supplementary Tables**

**Supplementary Table 1. Patient characteristics**

| **Characteristic** | **Total**  **(n=565)** | **Letermovir group**  **(n = 284)** | **Control group**  **(n = 281)** | ***P* value^1^** |
| --- | --- | --- | --- | --- |
| **Center,** ***n* (%)** |  |  |  | 0.0851 |
| Ruijin Hospital | 238 (42.1%) | 128 (45.1%) | 110 (39.1%) |  |
| Tongji Hospital | 224 (39.6%) | 114 (40.1%) | 110 (39.1%) |  |
| The First Affiliated Hospital of Zhejiang University School of Medicine | 103 (18.3%) | 42 (14.8%) | 61 (21.8%) |  |
| **Sex,** ***n* (%)** |  |  |  | 0.6742 |
| Male | 288 (51.0%) | 142 (50.0%) | 146 (48.0%) |  |
| Female | 277 (49.0%) | 142 (50.0%) | 135 (52.0%) |  |
| **Median age, years (range)** | 43 (18-70) | 42 (18-69) | 44 (18-70) | 0.8603 |
| **Disease type,** ***n* (%)** |  |  |  | 0.0866 |
| AA | 17 (3.0%) | 12 (4.2%) | 7 (1.8%) |  |
| ALL | 127 (22.5%) | 70 (24.6%) | 57 (20.3%) |  |
| AML | 327 (57.9%) | 153 (53.9%) | 174 (61.9%) |  |
| AUL | 2 (0.4%) | 2 (0.7%) | 0 (0%) |  |
| CMML | 3 (0.5%) | 2 (0.7%) | 1 (0.4%) |  |
| HL | 1 (0.2%) | 1 (0.4%) | 0 (0%) |  |
| MDS | 67 (11.9%) | 29 (10.2%) | 38 (13.5%) |  |
| MPAL | 9 (1.6%) | 7 (2.5%) | 2 (0.7%) |  |
| MS | 3 (0.5%) | 1 (0.4%) | 2 (0.7%) |  |
| NHL | 7 (1.2%) | 5 (1.8%) | 2 (0.7%) |  |
| PMF | 2 (0.3%) | 2 (0.6%) | 0 (0%) |  |
| **CMV serology,** ***n* (%)** |  |  |  | >0.999 |
| D+R+ | 561 (99.3%) | 282 (99.3%) | 279 (99.3%) |  |
| D-R+ | 4 (0.7%) | 2 (0.7%) | 2 (0.7%) |  |
| **EBV serology,** ***n* (%)** |  |  |  | 0.9057 |
| D+R+ | 562 (99.5%) | 283 (99.6%) | 279 (99.3%) |  |
| D+R- | 1 (0.2%) | 0 (0%) | 1 (0.4%) |  |
| D-R+ | 2 (0.3%) | 1 (0.4%) | 1 (0.4%) |  |
| D-R- | 0 (0%) | 0 (0%) | 0 (0%) |  |
| **Induction cycle,** ***n* (%)** |  |  |  |  |
| Evaluable patients^2^ | 465 | 232 | 233 | 0.1990 |
| ≤2 cycles | 433 (93.1%) | 221 (95.3%) | 212 (91.0%) |  |
| >2 cycles | 32 (6.9%) | 11 (4.7%) | 21 (9.0%) |  |
| **Consolidation cycle,** ***n* (%)** |  |  |  |  |
| Evaluable patients^2^ | 465 | 232 | 233 | 0.8941 |
| ≤2 cycles | 369 (79.4%) | 182 (78.4%) | 187 (80.3%) |  |
| >2 cycles | 96 (20.6%) | 50 (21.6%) | 46 (19.7%) |  |
| **HCT CI,** ***n* (%)** |  |  |  | 0.0911 |
| <3 | 547 (96.8%) | 271 (95.4%) | 276 (98.2%) |  |
| ≥3 | 18 (3.2%) | 13 (4.6%) | 5 (1.8%) |  |
| **Conditioning intensity,** ***n* (%)** |  |  |  | >0.999 |
| MAC | 476 (84.2%) | 239 (84.2%) | 237 (84.3%) |  |
| RIC | 89 (15.8%) | 45 (15.8%) | 44 (15.7%) |  |
| **ATG dose,** ***n* (%)** |  |  |  | 0.0347 |
| <5 mg/kg | 98 (17.3%) | 59 (20.8%) | 39 (13.9%) |  |
| ≥5 mg/kg | 467 (82.7%) | 225 (79.2%) | 242 (86.1%) |  |
| **Donor type,** ***n* (%)** |  |  |  | 0.0088 |
| MRD | 74 (13.1%) | 29 (10.2%) | 45 (16.0%) |  |
| MUD | 54 (9.6%) | 36 (12.7%) | 18 (6.4%) |  |
| HID | 437 (77.3%) | 219 (77.1%) | 218 (77.6%) |  |
| **CD34^+^ cell counts in graft, median (range, ×10^6^/kg)** | 8 (1.07-29.44) | 8.2 (1.83-25.3) | 7.56 (1.07-29.44) | 0.0025 |
| **cs-CMVi, *n* (%)** | 233 (41.2%) | 68 (23.9%) | 165 (68.7%) | <0.0001 |
| Median time from HCT to cs-CMVi (range, days) | 40 (13-336) | 104 (14-336) | 39 (13-330) | <0.0001 |
| **EBV infection,** ***n* (%)** | 316 (55.9%) | 166 (58.5%) | 150 (53.4%) | 0.9329 |
| **EBV DNAemia^3^, n (%)** | 266 (84.2%) | 133 (80.1%) | 133 (88.7%) |  |
| Median time from HCT to EBV DNAemia (range, days) | 42 (5-428) | 41 (5-397) | 42 (16-428) | 0.7387 |
| **EBV-disease, n (%)** | 21 (6.6%) | 10 (6.0%) | 11 (7.3%) |  |
| Median time from HCT to EBV disease (range, days) | 83 (23-806) | 95 (23-176) | 55 (24-806) | 0.3623 |
| **PTLD,** ***n* (%)** | 29 (9.2%) | 23 (13.9%) | 6 (4.0%) |  |
| Proven/probable | 14/15 | 9/14 | 5/1 | 0.0801 |
| Median time from HCT to PTLD (range, days) | 66 (21-452) | 66 (50-396) | 69.5 (21-452) | 0.5127 |
| **Other virus-related outcomes** |  |  |  |  |
| ADV, *n* (%) | 5 (0.9%) | 4 (1.4%) | 1 (0.4%) | 0.3728 |
| HHV-6B, *n* (%) | 35 (6.2%) | 18 (6.3%) | 17 (6.0%) | >0.999 |
| HSV-1/HSV-2, *n* (%) | 37 (6.5%) | 23 (8.1%) | 15 (5.0%) | 0.1732 |
| VZV, *n* (%) | 13 (2.3%) | 4 (1.4%) | 9 (3.2%) | 0.1726 |
| **aGvHD** |  |  |  |  |
| Grade I-IV | 246 (43.5%) | 121 (42.6%) | 125 (44.5%) | 0.6719 |
| Grade III-IV | 32 (5.7%) | 17 (6.0%) | 15 (5.3%) | 0.8560 |
| **Median follow-up (range, days)** | 390 (80-1239) | 284 (80-668) | 670 (105-1239) | <0.001 |

^1^ Fisher's exact test or Student’s t-test

^2^ Induction cycles and consolidation cycles were only evaluated for patients with AML, ALL, AUL and MPAL.

**Abbreviations:** AA, aplastic anaemia; ADV, adenovirus; aGvHD, acute graft versus host disease; ALL, acute lymphoblastic leukaemia; AML, acute myeloid leukaemia; ATG, anti-thymocyte globulin; AUL, acute undifferentiated leukaemia; CMML, chronic myelomonocytic leukaemia; CMV, cytomegalovirus; cs-CMVi: clinically significant CMV infection; EBV, Epstein-Barr virus; HCT-CI, hematopoietic cell transplantation-specific comorbidity index; HHV-6, human herpesvirus-6; HID, haploidentical donor; HL, Hodgkin's lymphoma; HSV, herpes simplex virus; MAC, myeloablative conditioning; MDS, myelodysplastic syndrome; MPAL, mixed phenotype acute leukaemia; MRD, matched related donor; MS, myeloid sarcoma; MUD, matched unrelated donor; NHL, non-Hodgkin's lymphoma; PMF, primary myelofibrosis; PTLD, post-transplant lymphoproliferative disorders; RIC, reduced-intensity conditioning; VZV, varicella-zoster virus.

**Supplementary Table 2. Clinical characteristics of patients with EBV-disease**

| **Case** | **Centre** | **Sex** | **Age** | **Disease** | **Donor type** | **Transplant date** | **Letermovir use** | **Antecedent EBV DNAemia** | **Time (days) from EBV disease to transplant** | **EBV positive pathological specimens** | **Diagnosis** | **Therapy** | **Response to therapy** | **Outcome** | **Cause of death** | **Follow-up time (days)** |
| --- | --- | --- | --- | --- | --- | --- | --- | --- | --- | --- | --- | --- | --- | --- | --- | --- |
| 1 | Ruijin hospital | M | 58 | AML | HID | 2021/7/8 | N | Y | 355 | BALF | Pneumonia | R*2 | CR | Live | / | 998 |
| 2 | Ruijin hospital | M | 32 | AML | HID | 2021/11/23 | N | Y | 83 | BALF | Pneumonia | R*2 | CR | Live | / | 860 |
| 3 | Ruijin hospital | F | 34 | ALL | HID | 2022/1/18 | N | Y | 51 | Gastrointestinal endoscopy for tissue biopsy | Gastroenteritis | R*4 | CR | Live | / | 804 |
| 4 | Ruijin hospital | M | 62 | AML | HID | 2022/10/31 | Y | Y | 39 | BALF | Pneumonia | R*3 | CR | Live | / | 518 |
| 5 | Ruijin hospital | F | 57 | AML | HID | 2022/11/9 | Y | Y | 23 | BALF | Pneumonia | R*3 | CR | Death | Infection | 229 |
| 6 | Ruijin hospital | M | 40 | AML | HID | 2023/3/14 | Y | Y | 176 | Cerebrospinal fluid | Encephalitis | R*2 | CR | Live | / | 384 |
| 7 | Ruijin hospital | M | 66 | AML | HID | 2023/5/31 | Y | Y | 60 |  | Hepatitis | R*2 | CR | Live | / | 306 |
| 8 | Ruijin hospital | M | 47 | AML | HID | 2023/8/16 | Y | Y | 91 | Cerebrospinal fluid | Encephalitis | R*1 | CR | Live | / | 229 |
| 9 | Tongji hospital | M | 33 | AML | HID | 2021/6/23 | N | Y | 806 | BALF | Pneumonia | R*2 | CR | Live | / | 875 |
| 10 | Tongji hospital | M | 32 | AML | HID | 2021/7/26 | N | Y | 326 | BALF | Pneumonia | R*2 | CR | Live | / | 887 |
| 11 | Tongji hospital | M | 43 | AML | HID | 2021/8/5 | N | Y | 83 | BALF | Pneumonia | R*2 | CR | Live | / | 865 |
| 12 | Tongji hospital | F | 41 | AML | MSD | 2022/4/14 | N | Y | 55 | BALF | Pneumonia | R*2 | CR | Live | / | 623 |
| 13 | Tongji hospital | F | 21 | AML | MUD | 2022/9/5 | Y | Y | 135 | BALF | Pneumonia | R*4 | CR | Live | / | 479 |
| 14 | Tongji hospital | F | 37 | MPAL | MMUD | 2023/1/16 | Y | Y | 99 | BALF | Pneumonia | R*3 | CR | Live | / | 346 |
| 15 | Tongji hospital | M | 39 | AML | HID | 2023/5/9 | Y | Y | 85 | Cerebrospinal fluid | Encephalitis | R*2 | CR | Live | / | 233 |
| 16 | Tongji hospital | M | 51 | AML | HID | 2023/6/29 | Y | Y | 175 | Gastrointestinal endoscopy for tissue biopsy | Gastroenteritis | R*2 | CR | Live | / | 193 |
| 17 | Tongji hospital | M | 42 | ALL | HID | 2023/6/30 | Y | Y | 105 | BALF | Pneumonia | R*2 | PD | Death | Pneumonia | 134 |
| 18 | The First Affiliated Hospital of Zhejiang University School of Medicine | M | 35 | AML | HID | 2021/8/6 | N | Y | 45 | Lymph node | Lymphadenitis | R*4+COP | CR | Live | / | 825 |
| 19 | The First Affiliated Hospital of Zhejiang University School of Medicine | M | 29 | AML | HID | 2021/8/19 | N | Y | 28 | Cerebrospinal fluid Cerebrospinal fluid | Encephalitis | R*4 | CR | Live | / | 945 |
| 20 | The First Affiliated Hospital of Zhejiang University School of Medicine | F | 26 | AML | HID | 2022/4/11 | N | Y | 32 | BALF | Pneumonia | R*2+COP | CR | Live | / | 645 |
| 21 | The First Affiliated Hospital of Zhejiang University School of Medicine | F | 19 | AML | HID | 2023/2/6 | N | Y | 24 | Cerebrospinal fluid | Encephalitis | R*4 | CR | Live | / | 409 |

**Abbreviations:** ALL, acute lymphoblastic leukaemia; AML, acute myeloid leukaemia; BALF, Bronchoalveolar Lavage Fluid; EBV, Epstein-Barr virus; EBER, Epstein-Barr early RNA; CHOPE, cyclophosphamide, doxorubicin, vincristine, prednisone and etoposide; CR, complete remission; CsA, Cyclosporin A; F, female; HID, haploidentical donor; ISH, in situ hybridization; M, male; MRD, matched related donor; MUD, matched unrelated donor; N, No; NA, not available; PD, progressive disease; R, Rituximab; Y, Yes.

**Supplementary Table 3. Clinical characteristics of patients with PTLD**

| **Case** | **Centre** | **Sex** | **Age** | **Disease** | **Donor type** | **Transplant date** | **Letermovir use** | **Antecedent EBV DNAemia** | **Time (days) from PTLD to transplant** | **The administration of IST when PTLD onset** | **Initial presentation** | | | | **PTLD category** | **Pathology** | | | | **Therapy for PTLD** | **Response to therapy** | **Outcome** | **Cause of death** | **Follow-up time (days)** |
| --- | --- | --- | --- | --- | --- | --- | --- | --- | --- | --- | --- | --- | --- | --- | --- | --- | --- | --- | --- | --- | --- | --- | --- | --- |
|  |  |  |  |  |  |  |  |  |  |  | **Fever** | **Lymphadenopathy** | **Hepatosplenomegaly** | **Other extranodal involvement** |  | **Pathology** | **Origin** | **EBER ISH** | **Ki-67** |  |  |  |  |  |
| 1 | Ruijin hospital | F | 53 | AML | HID | 2021/12/27 | N | Y | 83 | CsA 100mg/d | / | Y | / | / | ProvenPTLD | Polymorphic | Donor | Positive | 60% | R*4+CHOPE | CR | Alive | / | 826 |
| 2 | Ruijin hospital | M | 47 | ALL | HID | 2022/5/6 | N | Y | 60 | CsA 100mg/d | / | Y | / | / | ProvenPTLD | Polymorphic | Donor | Positive | 50% | R*4+CHOPE | CR | Alive | / | 696 |
| 3 | Ruijin hospital | M | 46 | ALL | **HID** | 2023/2/14 | Y | Y | 324 | CsA 100mg/d | / | Y | / | Lung | ProvenPTLD | Monomorphic | Donor | Positive | / | R*2+COP | CR | Alive | / | 412 |
| 4 | Ruijin hospital | F | 37 | AML | MUD | 2023/3/14 | Y | Y | 55 | CsA 100mg/d | Y | Y | / | / | Probable PTLD | / | / | / | / | R*2+COP | CR | Alive | / | 384 |
| 5 | Ruijin hospital | F | 54 | AML | HID | 2023/3/30 | Y | Y | 61 | CsA 100mg/d | / | Y | Y | Liver | Probable PTLD | / | / | / | / | R*4+COP | CR | Death | Infection | 90 |
| 6 | Ruijin hospital | F | 28 | ALL | HID | 2023/4/13 | Y | Y | 50 | CsA 100mg/d | / | Y | / | / | Probable PTLD | / | / | / | / | R*1+CHOP | CR | Alive | / | 354 |
| 7 | Ruijin hospital | F | 43 | AML | MUD | 2023/4/18 | Y | Y | 105 | CsA 100mg/d | / | Y | / | / | Probable PTLD | / | / | / | / | R*3+CHOP | CR | Alive | / | 347 |
| 8 | Ruijin hospital | F | 63 | ALL | HID | 2023/4/20 | Y | Y | 69 | CsA 100mg/d | / | Y | / | / | Probable PTLD | / | / | / | / | R*2+COP | CR | Alive | / | 349 |
| 9 | Ruijin hospital | F | 55 | AML | MUD | 2023/4/25 | Y | Y | 61 | CsA 100mg/d | Y | / | Y | Digestive tract | Proven PTLD | Polymorphic | Donor | Positive | 60% | R*4+CHOPE | CR | Alive | / | 342 |
| 10 | Ruijin hospital | M | 46 | ALL | HID | 2023/4/25 | Y | Y | 168 | CsA 100mg/d | / | Y | / | / | ProvenPTLD | Monomorphic | Donor | Positive | 70% | R*4+COP | In treatment | Alive | / | 342 |
| 11 | Ruijin hospital | F | 56 | AML | HID | 2023/4/27 | Y | Y | 62 | CsA 100mg/d | / | Y | / | / | Probable PTLD | / | / | / | / | R*4+COP | PD | Death | PTLD | 80 |
| 12 | Ruijin hospital | M | 51 | MPAL | HID | 2023/6/15 | Y | Y | 60 | CsA 100mg/d | / | Y | / | / | Probable PTLD | / | / | / | / | R*2+COPE | CR | Alive | / | 291 |
| 13 | Ruijin hospital | M | 42 | AML | HID | 2023/7/3 | Y | Y | 81 | CsA 100mg/d | / | Y | / | / | Probable PTLD | / | / | / | / | R*4+CHOPE | CR | Alive | / | 273 |
| 14 | Ruijin hospital | M | 18 | ALL | HID | 2023/7/19 | Y | Y | 51 | CsA 100mg/d | / | / | / | / | Probable PTLD | / | / | / | / | R*2+COP | CR | Alive | / | 257 |
| 15 | Ruijin hospital | M | 33 | AML | HID | 2023/9/21 | Y | Y | 396 | CsA 100mg/d | / | Y | / | / | Proven PTLD | Monomorphic | Donor | Positive | 60% | R*2+COP | In treatment | Alive | / | 193 |
| 16 | Ruijin hospital | F | 31 | AML | HID | 2023/12/21 | Y | Y | 66 | CsA 100mg/d | / | Y | / | / | Proven PTLD | Monomorphic | Donor | Positive | 40% | R*2+CHOPE | CR | Alive | / | 102 |
| 17 | Ruijin hospital | F | 28 | ALL | HID | 2023/12/30 | Y | Y | 56 | CsA 100mg/d | / | Y | / | / | Proven PTLD | Monomorphic | Donor | Positive | 60% | R*4+COPE | CR | Alive | / | 90 |
| 18 | Tongji hospital | M | 42 | AML | HID | 2022/10/9 | Y | Y | 87 | CsA 100mg/d | Y | / | / | Lung | Proven PTLD | / | Donor | Positive | / | R*4+CHOPE | CR | Death | Infection | 343 |
| 19 | Tongji hospital | F | 26 | AML | HID | 2021/1/25 | N | Y | 21 | / | / | / | / | / | Probable PTLD | / | / | / | / | R+MTX | PD | Death | PTLD | 145 |
| 20 | Tongji hospital | M | 33 | AML | HID | 2021/11/8 | N | Y | 24 | MMF 500mg/d+CsA 125mg/d | Y | Y | / | Stomach，Muscle | Proven PTLD | Polymorphic | Donor | Positive | 50% | R*4 | CR | Death | Relapse | 144 |
| 21 | Tongji hospital | M | 24 | AML | HID | 2021/11/25 | N | Y | 79 | CsA 75mg/d | Y | Y | / | / | Proven PTLD | Polymorphic | Donor | Positive | 90% | Venetoclax+R*1+COP | CR | Death | Infection | 124 |
| 22 | Tongji hospital | F | 47 | AML | MRD | 2022/1/29 | N | Y | 452 | / |  | Y | / | / | Probable PTLD | / | / | / | / | R*2+COP | CR | Alive | / | 698 |
| 23 | Tongji hospital | F | 18 | AML | HID | 2023/2/12 | Y | Y | 93 | CsA 100mg/d | Y | Y | / | / | Probable PTLD | / | / | / | / | IST reduction | CR | Alive | / | 319 |
| 24 | Tongji hospital | F | 32 | AML | HID | 2023/4/10 | Y | Y | 91 | CsA 100mg/d | / | Y | / | / | Proven PTLD | Monomorphic | Donor | Positive | 80% | R*2+COP+IST reduction | CR | Alive | / | 262 |
| 25 | Tongji hospital | F | 22 | ALL | HID | 2023/4/25 | Y | Y | 51 | / | Y | Y | / | / | Probable PTLD | / | / | / | / | / | / | Alive | / | 247 |
| 26 | Tongji hospital | F | 32 | AML | HID | 2023/9/8 | Y | Y | 112 | / | Y | Y | / | / | Probable PTLD | / | / | / | / | R*2 | PD | Death | PTLD | 206 |
| 27 | Tongji hospital | F | 51 | AA | MRD | 2023/10/24 | Y | Y | 59 | CsA 75mg/d | / | Y | / | / | Proven PTLD | Polymorphic | Donor | Positive | 60% | R*2+COPE | CR | Alive | / | 160 |
| 28 | The First Affiliated Hospital of Zhejiang University School of Medicine | M | 37 | AML | MUD | 2023/3/6 | Y | Y | 86 | MMF 250 mg/d + CsA 50mg/d | / | Y | / | Oral mucosa， Skin | Proven PTLD | Polymorphic | Donor | Positive | 60% | R*2+CHOPE | CR | Alive | / | 248 |
| 29 | The First Affiliated Hospital of Zhejiang University School of Medicine | F | 48 | AML | HID | 2023/7/28 | Y | Y | 57 | CsA 100mg/d | / | Y | / | / | Probable PTLD | / | / | / | / | R*2+CHOPE | CR | Alive | / | 368 |

**Abbreviations:** AA, aplastic anaemia; ALL, acute lymphoblastic leukaemia; AML, acute myeloid leukaemia; EBV, Epstein-Barr virus; EBER, Epstein-Barr early RNA; CHOPE, cyclophosphamide, doxorubicin, vincristine, prednisone and etoposide; CR, complete remission; CsA, Cyclosporin A; ETP, early T-cell precursor acute lymphoblastic leukaemia; F, female; HID, haploidentical donor; ISH, in situ hybridization; IST, immunosuppressive therapy; M, male; MRD, matched related donor; MTX, methotrexatel; MUD, matched unrelated donor; N, No; NA, not available; PD, progressive disease; PTLD, post-transplant lymphoproliferative disorder; R, Rituximab; Y, Y

**References**

1. Faye A, Van Den Abeele T, Peuchmaur M, Mathieu-Boue A, Vilmer E. Anti-CD20 monoclonal antibody for post-transplant lymphoproliferative disorders. Lancet. 1998;352(9136):1285.

2. Coppoletta S, Tedone E, Galano B, Soracco M, Raiola AM, Lamparelli T, et al. Rituximab treatment for Epstein-Barr virus DNAemia after alternative-donor hematopoietic stem cell transplantation. Biol Blood Marrow Transplant. 2011;17(6):901-7.

3. Liu L, Ji X, Zhu P, Yang L, Shi J, Zhao Y, et al. Double filtration plasmapheresis combined with rituximab for donor-specific antibody desensitization in haploidentical haematopoietic stem cell transplantation. Br J Haematol. 2023;203(5):829-39.

4. Landgren O, Gilbert ES, Rizzo JD, Socié G, Banks PM, Sobocinski KA, et al. Risk factors for lymphoproliferative disorders after allogeneic hematopoietic cell transplantation. Blood. 2009;113(20):4992-5001.

5. Jiang JL, Gao WH, Wang LN, Wan M, Wang L, Hu J. Low Incidence of Relapse with a Moderate Conditioning Regimen of Fludarabine, Busulfan, and Melphalan for Patients with Myeloid Malignancies: A Single-Center Analysis of 100 Patients. Transplant Cell Ther. 2023;29(8):512 e1- e8.

6. Wang Y, Liu Q-F, Lin R, Yang T, Xu Y-J, Mo X-D, et al. Optimizing antithymocyte globulin dosing in haploidentical hematopoietic cell transplantation: long-term follow-up of a multicenter, randomized controlled trial. Sci Bull (Beijing). 2021;66(24):2498-505.

7. Wang Y, Liu Q-F, Xu L-P, Liu K-Y, Zhang X-H, Ma X, et al. Haploidentical vs identical-sibling transplant for AML in remission: a multicenter, prospective study. Blood. 2015;125(25):3956-62.

8. Wang L, Gao W, Wang L, Wan M, Jiang J, Hu J. Cladribine-based debulking chemotherapy sequential with reduced intensity regimen and post-transplantation maintenance for refractory myeloid leukemia: a prospective phase II clinical trial. Bone Marrow Transplant. 2022;57(6):1004-6.

9. Xu X, Yang J, Cai Y, Li S, Niu J, Zhou K, et al. Low dose anti-thymocyte globulin with low dose posttransplant cyclophosphamide (low dose ATG/PTCy) can reduce the risk of graft-versus-host disease as compared with standard-dose anti-thymocyte globulin in haploidentical peripheral hematopoietic stem cell transplantation combined with unrelated cord blood. Bone Marrow Transplant. 2021;56(3):705-8.

10. Huang J, Shi B, Yu S, Xue M, Wang L, Jiang J, et al. Efficacy of blinatumomab as maintenance therapy for B-lineage acute lymphoblastic leukemia/lymphoma following allogeneic hematopoietic cell transplantation. Blood Cancer J. 2024;14(1):109.

11. Tong Y, Pang XL, Mabilangan C, Preiksaitis JK. Determination of the Biological Form of Human Cytomegalovirus DNA in the Plasma of Solid-Organ Transplant Recipients. J Infect Dis. 2017;215(7):1094-101.

12. Kwong YL, Pang AWK, Leung AYH, Chim CS, Tse E. Quantification of circulating Epstein-Barr virus DNA in NK/T-cell lymphoma treated with the SMILE protocol: diagnostic and prognostic significance. Leukemia. 2014;28(4):865-70.

13. Au W-Y, Pang A, Choy C, Chim C-S, Kwong Y-L. Quantification of circulating Epstein-Barr virus (EBV) DNA in the diagnosis and monitoring of natural killer cell and EBV-positive lymphomas in immunocompetent patients. Blood. 2004;104(1):243-9.

14. Alaggio R, Amador C, Anagnostopoulos I, Attygalle AD, de Oliveira Araujo IB, Berti E, et al. Correction: "The 5th edition of The World Health Organization Classification of Haematolymphoid Tumours: Lymphoid Neoplasms" Leukemia. 2022 Jul;36(7):1720-1748. Leukemia. 2023;37(9):1944-51.

15. Styczynski J, van der Velden W, Fox CP, Engelhard D, de la Camara R, Cordonnier C, et al. Management of Epstein-Barr Virus infections and post-transplant lymphoproliferative disorders in patients after allogeneic hematopoietic stem cell transplantation: Sixth European Conference on Infections in Leukemia (ECIL-6) guidelines. Haematologica. 2016;101(7):803-11.

16. Styczynski J, Reusser P, Einsele H, de la Camara R, Cordonnier C, Ward KN, et al. Management of HSV, VZV and EBV infections in patients with hematological malignancies and after SCT: guidelines from the Second European Conference on Infections in Leukemia. Bone Marrow Transplant. 2009;43(10):757-70.

17. Tellez J, Jaing C, Wang J, Green R, Chen M. Detection of Epstein-Barr virus (EBV) in human lymphoma tissue by a novel microbial detection array. Biomark Res. 2014;2(1):24.

18. Ullmann AJ, Schmidt-Hieber M, Bertz H, Heinz WJ, Kiehl M, Krüger W, et al. Infectious diseases in allogeneic haematopoietic stem cell transplantation: prevention and prophylaxis strategy guidelines 2016. Annals of Hematology. 2016;95(9):1435-55.

19. Tomblyn M, Chiller T, Einsele H, Gress R, Sepkowitz K, Storek J, et al. Guidelines for preventing infectious complications among hematopoietic cell transplantation recipients: a global perspective. Biol Blood Marrow Transplant. 2009;15(10):1143-238.

20. Henze L, Buhl C, Sandherr M, Cornely OA, Heinz WJ, Khodamoradi Y, et al. Management of herpesvirus reactivations in patients with solid tumours and hematologic malignancies: update of the Guidelines of the Infectious Diseases Working Party (AGIHO) of the German Society for Hematology and Medical Oncology (DGHO) on herpes simplex virus type 1, herpes simplex virus type 2, and varicella zoster virus. Annals of Hematology. 2022;101(3):491-511.

21. Ye Q, Wang J, Chen M, Nie W, Zhang H, Su X, et al. Interferon-gamma FlowSpot assay for the measurement of the T-cell response to cytomegalovirus. Heliyon. 2023;9(6):e16792.
